# Memoir on the Pearly Nautilus

**Published:** 1833-01-01

**Authors:** 


**Memoir on the Pearly Nautilus, (N. Pompilius Linnæus.) By *Richard Owen*, Esq. M.R.C.S., Z.S. &c. &c. Quarto, pp. 68. Plates, 8. London, 1832.**

(Published by direction of the Council of the Royal College of Surgeons in London.)

This is one of the most valuable and important monographs on any subject of natural history which for many years has been published; and we particularly wish the attention of our readers to be directed to it, not only from the admirable details of the memoir itself, but also as it affords a pleasing evidence how easily many of the medical profession may contribute largely and efficiently to the promotion of other sciences as well as of their own. When we state that the animal, whose anatomy Mr. Owen has here so beautifully developed, though abounding in many seas, has been long coveted, as perhaps the most rare of all prizes by naturalists; (for, judging from the records of books, it has been only twice seen and described, before the present specimen was obtained, namely, by Aristotle, and by the Dutch professor Ramphius, who lived nearly a century ago;) we hope that it will be an incentive to such surgeons as visit foreign climates, diligently to study the departments of zoology. We are indebted for the specimen to Mr. George Bennett, who during a recent voyage among the Polynesian islands was indefatigable in collecting materials of every kind for the advancement of knowledge. The circumstances connected with its capture are thus related in his journal.

"*Island of Erromanga, New Hebrides, August 24th,* 1829.

"In the evening a pearly nautilus (nautilus pompilius of Linnæus) was seen in Marekini Bay, floating on the surface of the water, and resembling, as the sailors expressed it, a dead tortoiseshell cat. It was captured, but not before the upper part of the shell had been broken by the boat-hook in the eagerness to take it, as the animal was sinking when caught. On its being brought on board, my attention was directed to possessing the inhabitant, which I succeeded in procuring. I immediately detached the animal from the fractured portions of the shell, to which it is attached by two oval muscular attachments, one on each side, and put it into spirits. The animal kept the tentacula closely contracted, and the only evidence of vitality was a slight contractile motion of the body. On laying carefully open that portion of the shell, which contains the chambers, it was found to contain water."

The position of the nautilus in the Cuvierian arrangement of natural history is among the cephalopodous mollusca, which most of our readers probably know, are the most perfectly organized of invertebrate animals. The appellation is derived from their having long, fleshy productions or tentacula issuing from the *head*, and supplying the place of *feet*, and we may add, also of hands. There are few individuals known, viz. eight or nine species of the cuttle-fish, including the sepiæ, loligo, and the octopies; two species of nautilus, and one species of argonauta. The shells of the two last genera are sufficiently common, and are universally admired, and receive the vulgar names of the pearly and of the paper sailor.

The cause of the extreme rarity of finding what is ordinarily called a living shell, that is, with the animal inclosed, of the former, is, that it is almost always sheltered in the depths of the ocean, and seldom comes to the surface. We cannot afford space for any minute details, but shall allude only to the more curious and interesting particulars of its structure. The nautilus has an exterior investment, or mantle as it is termed, somewhat similar to what is seen in the common cuttle-fish—it is much thicker and stronger anteriorly than posteriorly, where it is altogether inclosed in, and and thus protected by, the shell. From the extreme posterior point of this sac is continued a small tubular membranous process, which passes through the syphonic apertures in the septa of the chambers of the shell, probably to the innermost one. In Mr. Owen's specimen, it had unfortunately been broken off short; an artery and vein are included within its thin external membrane, but how they are distributed remains unknown. From the head proceed forty digitations or hollow processes which contain each an annulated cirrus, or tentacle; these tentacles are longer than the canals in which they are lodged, and appear to possess considerable projectile, and retractile powers. There is not the slightest appearance of acetabula or suckers on the surface of the processes; the eyes are about the size of hazle-nuts; they are not contained in orbits, but are simply attached to the sheath or mantle behind the processes. The mouth is completely concealed among numerous tentacula; on separating these, the organs of mastication were found to consist, as in the cuttle-fish, of two strong hooked mandibles, like the bill of a parrot. The internal skeleton or frame-work, from which the principal masses of muscle take their origin, is soft and cartilaginous, resembling much the cartilage of the skeleton of a skate. It is less perfect superiorly than we find in the sepia; the circle being incomplete behind, thus leaving the brain to be protected only by its membranous sheath.

To recur to the two mandibles, we find that their extremities are of a calcareous nature, and of a hardness apparently adequate to break through the densest crustaceous coverings, or even shells of moderate thickness; the margins are dentated, and thus probably designed to act upon hard substances, while the sharp edges of the beak of the cuttle-fish better adapt it for cutting and lacerating the soft bodies of fish. What adds much to the interest of this examination is the light which it throws on some organic remains, which were formerly considered to be the fossil beaks of birds; but which, no doubt appertain to the fossil shells of the large nautili met with, in the same strata.

The tongue of the nautilus is large, and has all the characters of a perfect organ of taste. The œsophagus is three-fourths of an inch long, and dilates into a capacious pouch, or crop, which opens by a short canal, into an oval gizzard, and this into the intestinal tube, which terminates between the bronchiæ. The whole of the alimentary canal was filled with the fragments of crustaceous animals. The liver is large and bulky; there is not any trace of structure analogous to the ink-bag of the cuttle-fish.

The respiratory and circulatory organs present some well-marked and characteristic peculiarities by which the nautilus is distinguished from the other cephalopods. It has four bronchiæ, whereas *they* have only two; and the bronchial arteries arise directly from the central venous sinus or dilatation of the vena cava, without the interposition of any ventricle or bronchial heart, as in the higher cephalopods; one, among many other beautiful illustrations, of the law of animal development, that the perfection and extent of the aërating functions is, as it were, a test, or measure of the muscular activities of the creature. The nautilus has much less power of loco-motion than the cuttle-fish, in consequence of the inferior energy of the bronchial circulation, arising from the deficiency of a bronchial ventricle. The vena cava is very powerfully muscular; and between the fibres Mr. Owen discovered about fifteen round apertures in the membrane of the vein, and in the corresponding part of the peritoneum, so that the latter membrane is continuous with the lining membrane of the vein; and thus the blood may pass into the general abdominal cavity, and the fluid contents of that cavity be reciprocally received or absorbed into the vein. That great anatomist whom science has recently lost, Baron Cuvier, first detected a similar mechanism in the genus aplysia; and hence he was led to infer that the absorbent system ceases entirely in the mollusks, and "*a fortiori,*" in the animals below them in the scale.

The nervous system of the pearly nautilus, though analogous, is in many respects inferior to that of other cephalopods; the eye is also much more simple; the pupil is very small; the lens is probably wanting, and the retina, as well as the rest of the interior of the cavity of the eye-ball, is covered by a black pigment, which is consequently here, as in the cuttle-fish, interposed between the impinging rays of light and the sentient membrane.

No distinct organ for hearing could be detected. The organs of generation in the present specimen were those of the female, and consisted of an ovary, an oviduct, and of an accessory glandular apparatus.

The chambered structure of the shell is indicative of the animal during its growth, having periodically divested its mansion, for another and a larger one; had the whole of the chambers been filled up with calcareous matter, the shell must have become so cumbersome, as to be incompatible with the locomotive faculties of its inhabitant. The exact use of the posterior membranous tube and its relation to the siphuncular apertures of the septa and chambers is still problematical. Dr. Hooke's theory was, that by means of it the animal has the power of generating air and expelling it into the deserted chambers, somewhat after the fashion of a fish's air-bladder, in order to render its specific gravity less, when it wishes to rise to the surface.

From a very careful examination of the anatomy of the nautilus Mr. Owen is led to consider it as "an osculant," or connecting form between the cephalopodous and the gasteropodous mollusca; and he has made it the type of the second order of the cephalopod, in which order he proposes to include such as have four bronchiæ. In the first order, viz. Dibronchiata, are arranged the sepia, octopus, loligo, ocythoë, or argonauta, &c.; while in the second, or Tetrabronchiata, he places the nautilus, spirula (?), and various fossil shells, as the bilemnites, ammonites, orbulites, &c. &c. But to such of our readers as have a taste for natural history, we must advise them not to be satisfied with the preceding notice, but consult and study the orginal, which is altogether a masterly specimen of genuine philosophic inquiry directed to anatomical and physiological pursuits. We hope that many will imitate the zeal of Mr. Bennett, and that we may again soon meet Mr. Owen in the field of his interesting labours.

---

